# Antibacterial effect of Manuka honey on *Clostridium difficile*

**DOI:** 10.1186/1756-0500-6-188

**Published:** 2013-05-07

**Authors:** Eric N Hammond, Eric S Donkor

**Affiliations:** 1 Global Health Systems Solutions, Accra, Ghana; 2 Department of Microbiology, University of Ghana Medical School, Accra, Ghana; 3 Department of Microbiology, University of Wales Institute Cardiff, Cardiff, UK

## Abstract

**Background:**

Manuka honey originates from the manuka tree (*Leptospermum scoparium*) and its antimicrobial effect has been attributed to a property referred to as Unique Manuka Factor that is absent in other types of honey. Antibacterial activity of Manuka honey has been documented for several bacterial pathogens, however there is no information on *Clostridium difficile*, an important nosocomial pathogen. In this study we investigated susceptibility of *C*. *difficile* to Manuka honey and whether the activity is bactericidal or bacteriostatic.

**Methods:**

Three *C*. *difficile* strains were subjected to the broth dilution method to determine minimum inhibitory concentrations (MIC) and minimum bactericidal concentrations (MBC) for Manuka honey. The agar well diffusion method was also used to investigate sensitivity of the *C*. *difficile* strains to Manuka honey.

**Results:**

The MIC values of the three *C*. *difficile* strains were the same (6.25% v/v). Similarly, MBC values of the three *C*. *difficile* strains were the same (6.25% v/v). The activity of Manuka honey against all three *C*. *difficile* strains was bactericidal. A dose–response relationship was observed between the concentrations of Manuka honey and zones of inhibition formed by the *C*. *difficile* strains, in which increasing concentrations of Manuka honey resulted in increasing size of zone of inhibition formed. Maximum zone of inhibition was observed at 50% (v/v) Manuka honey and the growth inhibition persisted over 7 days.

**Conclusion:**

*C*. *difficile* is appreciably susceptible to Manuka honey and this may offer an effective way of treating infections caused by the organism.

## Background

*Clostridium difficile* is a Gram positive anaerobic spore-forming bacillus, and is part of the normal gut flora in less than 5% of humans [1]. The organism is associated with severe infections including diarrhea, pseudomembranous colitis, toxic megacolon, perforation of the colon, and in some cases, sepsis [2]. *C*. *difficile* is an important nosocomial agent and currently accounts for 30-50% of hospital acquired infections with serious economic burden for many countries [3,4]. A number of risk factors for *C*. *difficile* associated diseases, including the use of certain antibiotics, particularly fluoroquinolones, have been identified [4-6]. In the pathogenesis of diarrhoea caused by the organism, these antibiotics suppress normal flora of the gut and allow the proliferation of *C*. *difficile* with the production of two toxins (TcdA and TcdB) which cause the disease [6,7].

Antibiotic resistance is a major public health threat especially, with important pathogens such as *C*. *difficile*[8]. The problem is associated with overuse and misuse of antibiotics that provide selective pressure favouring the emergence of resistant strains [9,10]. The escalating trend of microbial resistance to essential antibiotics, especially multidrug resistance underscores the need for evaluating alternative potential therapeutic agents with antibacterial properties. The use of honey for treating microbial infections dates back to ancient times, though antimicrobial properties of Manuka honey was discovered recently [11-16]. Manuka honey originates from the manuka tree (*Leptospermum scoparium*) and its antimicrobial effect has been attributed to a property referred to as Unique Manuka Factor that is absent in other types of honey [17]. Lately, studies have shown that the active ingredient in Manuka honey is Methylglyoxal [18,19], and this compound is known to have synergistic effect with some antibiotics such as piperacillin [20]. To date there are numerous studies that have demonstrated the therapeutic properties of Manuka honey, and have confirmed its activity against a wide range of pathogenic bacteria [21-23]. Consequently, Manuka honey has been recommended for the treatment of ailments such as leg ulcers, pilonidal sinus disease and gastrointestinal infection [24,25]. Though susceptibility of several bacterial pathogens to Manuka honey has been investigated, there is no data on *C*. *difficile*, and hence the current study investigated the antibacterial effect of Manuka honey against the organism. In this study, we provide evidence of the susceptibility of *C*. *difficile* to Manuka honey and that Manuka honey is bactericidal.

## Methods

### *C*. *difficile* strains

Three *C*. *difficile* strains were used in this study. The three strains were labeled Strains A, B and C. Strain A was the ATCC 9689 strain (PCR-ribotype X). Strains B and C were clinical isolates of PCR ribotypes 027 and 106 respectively. The strains were provided by the Anaerobe Reference Laboratory at the University of Wales Hospital, and maintained at the Department of Microbiology, University of Wales Institute Cardiff (UWIC). The *C*. *difficile* strains were grown/stored in Robertson’s Cook meat medium (Oxoid, Cambridge, UK) and a purity test [26] was performed on each strain before it was used in the study.

### Manuka honey

Woundcare™ 18+ Active Manuka honey (potency equivalent of greater than 18% v/v phenol) with non-peroxide antibacterial activity from Comvita UK was used in this study.

#### Determination of MIC/MBC of Manuka honey for *C*. *difficile* strains by broth dilution

Minimum inhibitory concentrations (MIC) refers to the lowest concentration of an antimicrobial that will inhibit the visible growth of a microorganism while minimum bactericidal concentration (MBC) refers to lowest concentration of an antimicrobial that will kill the microorganism [26,27]. MICs of Manuka honey for the *C*. *difficile* strains were determined using the broth dilution method of susceptibility testing described by European Committee on Antimicrobial Susceptibility Testing [18]. A stock solution of 50% (v/v) Manuka honey was prepared by dissolving 12.5 g honey in 25ml sterile deionised water. Four millilitres of this solution was pipetted into an empty test tube and labelled 1. Two millilitres of prereduced thioglycolate broth (Oxoid, Cambridge, UK) was pipetted into nine other test tubes. Subsequently 2 ml honey solution from test tube 1 was transferred to test tube 2 containing thioglycolate to prepare a two-fold serial dilution. The tubes were inoculated with 100 μl (10^5^ cfu) of an overnight culture of a *C*. *difficile* strain in an anaerobic cabinet (Don Whitley Scientific/MACS, UK) at 37°C for 48 hours. Positive and negative controls were set with 2 mls of the thioglycolate broth (without honey solution) containing 100 μl (10^5^ cfu) inoculums and 2 ml of the thioglycolate broth without inoculum respectively. After 48 hours incubation, each tube was examined for the presence and absence of turbidity to indicate growth of the microorganism. The first broth or lowest concentration of honey that inhibited growth of the microorganism was designated the MIC [26,27]. The results were scored as ‘bacterial growth’ (+) and ‘no bacterial growth’ (−). This test was done in triplicate to ensure reproducibility of results.

To determine the MBC, 10 μl of a sample from the MIC broth that showed no turbidity was streaked onto drug-free medium, prereduced fastidious anaerobic agar plates (Oxoid, Cambridge, UK) in an anaerobic cabinet and incubated at 37°C for 24 hours. MBC was defined as the first dilution at which no growth was examined [27]. Any colonies that developed were scored as ‘bacterial growth’ (+) and ‘no bacterial growth’ (−).

### Evaluation of sensitivity of *C*. *difficile* strains to Manuka honey by agar diffusion

Sensitivity of the *C*. *difficile* strains to Manuka honey was determined using the agar diffusion method of susceptibility testing described by European Committee on Antimicrobial Susceptibility Testing [28]. An overnight culture of the test strain in Robertson’s Cook Meat medium (Oxoid, Cambridge, UK) was used to prepare a lawn on a prereduced Fastidious anaerobic agar plate by uniformly swabbing the surface of the agar with a sterile swab stick dipped into the broth culture. Wells were then cut in each agar plate aseptically using a sterile cork borer (8 mm in diameter). These wells were subsequently filled with 350 μl of 50% (v/v) honey solution (5 g honey dissolved in double strength iso-sensitest broth and made up to the 10 ml mark) and incubated at 37°C for 7 days in an anaerobic cabinet. The negative control used in this experiment consisted of 350 μl double strength iso-sensitest broth (Oxoid, Cambridge, UK) without honey. The zones of inhibition were measured every 24 hours over a period of 7 days using digital vernier callipers (Swiss Precision/Digimax) and compared to the readings of the control plate. Incubated plates showing zones of inhibition were also monitored from days 1 to 7 for the appearance of *C*. *difficile* colonies in the zone of inhibition. For each *C*. *difficile* strain, the sensitivity experiments were performed for honey solutions of 40%, 30%, 20% and 10% (v/v) in triplicates. However, the plates were incubated up to 48 hours (2 days), as the inhibition zones of the 50% v/v Manuka honey did not change after 48 hours of incubation.

## Results and discussion

The MIC and MBC of Manuka honey for the three *C*. *difficile* strains investigated is shown in Table 1. The MIC values of the three *C*. *difficile* strains were the same (6.25% v/v). Similarly, MBC values of the three *C*. *difficile* strains were the same (6.25% v/v). The MBC/MIC ratio for each of the strains was 1.0. MIC/MBC are used in confirming susceptibility test results, especially for serious infections, and are also important for monitoring the activity of new antimicrobial agents [27,29]. Though there is hardly any data on antibacterial effect of Manuka honey against *C*. *difficile*, MIC and MBC values of several bacterial agents in relation to Manuka honey have been determined and can be used for comparison with our data. Cooper *et al*. [30] reported that the MIC of 58 isolates of *Staphylococcus aureus* from infected wound was 3 - 4% (v/v). In another report, Cooper and Molan [31] determined the MIC of Manuka honey for 20 strains of *Pseudomonas aeruginosa* isolated from infected wounds to be between 5.5% (v/v) and 8.7% (v/v). Furthermore, Cooper *et al*. [32] tested the sensitivity of 17 strains of *P*. *aeruginosa* isolated from infected burns with Manuka honey (with median level of activity) and observed MICs below 10% (v/v) for all the test strains. Wilkinson and Cavanagh [33] reported that Manuka honey was effective against many organisms including *S*. *aureus* (MIC_100_ = 1.8), *E*. *coli* (MIC_100_ = 3.7), *Salmonella typhimurium* (MIC_100_ = 6) and *Proteus mirabilis* (MIC_100_ = 7.3) % (v/v). The MIC values observed for the *C*. *difficile* strains in this study thus appear to be similar to MIC values that have been reported for some other bacteria particularly, *P*. *aeruginosa*. It is known that bacteriostatic antimicrobial agents have an MBC/MIC ratio greater than or equal to 16 for a given bacterium, while for bactericidal antimicrobial agents the MBC/MIC ratio is less than or equal to 4 [34]. The MBC/MIC ratios for the different *C*. *difficile* strains in this study (Table 1) suggest that Manuka honey exhibits a bactericidal mode of action against *C*. *difficile*.

**Table 1:** **Minimum inhibitory concentrations and minimum bactericidal concentrations of Manuka honey for different *C*. *difficile *strains**

**C. difficile strain**	**PCR ribotype**	**MIC**	**MBC**	**MBC/MIC ratio**
Strain A (ATCC 9689)	PCR ribotype X	6.25	6.25	1
Strain B	PCR ribotype 027	6.25	6.25	1
Strain C	PCR ribotype 106	6.25	6.25	1

**Figure 1:**
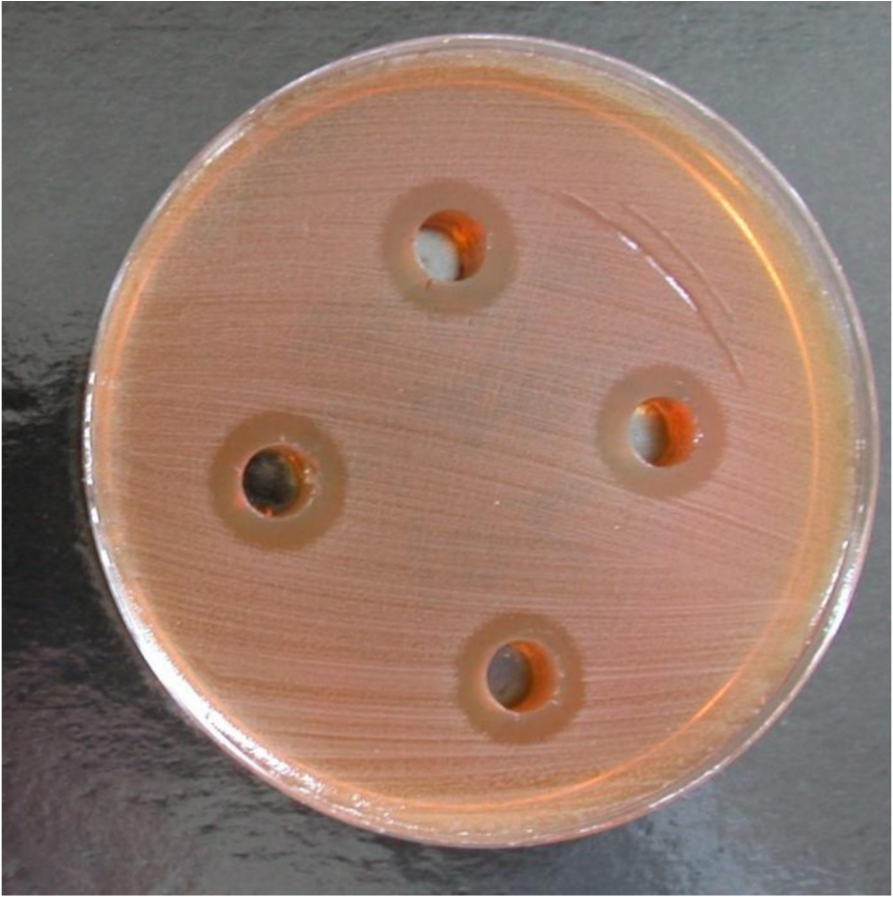
**Picture showing inhibition zones after 7 days incubation of *Clostridium difficile *in the presence of 50% v/v Manuka honey.**

Further evidence of the efficacy of Manuka honey against the *C*. *difficile* test strains was determined by measuring zone of inhibition in agar well diffusion experiments (Table 2/Figure 1). It was observed that 10 and 20% (v/v) Manuka honey concentrations did not show any visible measureable zone of inhibition indicating that at these concentrations, the organisms were not sensitive to the effect of Manuka honey. At 30% (v/v) Manuka honey concentration, there was no visible effect on Strain C, however zones of inhibition were observed for Strain A (ATCC 9689) and Strain B, with the former being more sensitive. At concentrations of 40 and 50% (v/v), all the three *C*. *difficile* test strains showed considerable sensitivity to Manuka honey and produced comparable zones of inhibition. Generally, in this study, increasing Manuka honey concentrations correlated with increase in the size of zone of inhibition and is due to high quantity of antibacterial properties such as Unique Manuka Factor present in Manuka honey as well as its osmolarity which increases with increasing honey concentration [17]. Generally, the test strains were inhibited at lower Manuka honey concentrations in liquid medium (Table 1) than on agar well diffusion plates (Table 2) which may be due to the Manuka honey solution being able to diffuse more uniformly, efficiently and faster in the former.

**Table 2:** **Sensitivity of *C*. *difficile *strains to Manuka honey by agar diffusion**

**Day**	**Honey concentration (% v/v)**	**Mean zone of inhibition (mm) ± SD**		
		**Strain A (ATCC 9689)**	**Strain B**	**Strain C**
1-7	0	0	0	0
1-7	10	0	0	0
1-7	20	0	0	0
1	30	0	9.1 ± 0.12	0
2	30	8.4 ± 0.11	9.3 ± 0.29	0
1	40	9.5 ± 0.31	10.2 ± 0.33	10.2 ± 0.27
2	40	10.2 ± 0.4	10.4 ± 0.20	10.2 ± 0.24
1	50	13.6 ± 0.78	13.9 ± 0.32	14.0 ± 0.24
2	50	14.3 ± 0.50	14.5 ± 0.22	14.2 ± 0.23
3	50	13.9 ± 0.50	14.0 ± 0.28	13.9 ± 0.26
4	50	14.2 ± 0.63	14.1 ± 0.42	14.2 ± 0.28
7	50	14.5 ± 0.52	14.5 ± 0.41	14.7 ± 0.45

± SD (standard deviation).

## Conclusion

In this study, we provide the first data on antibacterial effect of Manuka honey against *C*. *difficile*. Our data demonstrates susceptibility of the *C*. *difficile* strains to Manuka honey with MIC of 6.25% (v/v) and MBC of 6.25% (v/v). Manuka honey exhibits a bactericidal action against *C*. *difficile*, a feature which is likely to make Manuka honey highly attractive in the treatment of bacterial infections. Our data adds to the body of research evidence in support of the broad antibacterial spectrum of Manuka honey.

## Competing interests

The authors declare that they have no competing interests.

## Authors’ contributions

Laboratory experiments were carried out by ENH. Interpretation of the data was done by ENH and ESD. The manuscript was written by ESD and ENH. Both authors read and approved the final manuscript.
